# A Novel Flavonoid Kushenol Z from *Sophora flavescens* Mediates mTOR Pathway by Inhibiting Phosphodiesterase and Akt Activity to Induce Apoptosis in Non-Small-Cell Lung Cancer Cells

**DOI:** 10.3390/molecules24244425

**Published:** 2019-12-04

**Authors:** Hao Chen, Jie Yang, Ji Hao, Yibing Lv, Lu Chen, Qinxiong Lin, Jingquan Yuan, Xinzhou Yang

**Affiliations:** 1 Guangxi Scientific Research Center of Traditional Chinese Medicine, Guangxi University of Chinese Medicine, Nanning 530001, China; 348734573@163.com; 2 School of Pharmaceutical Sciences, South-Central University for Nationalities, Wuhan 430074, China; 15527732263@163.com (J.Y.); 13618615220@163.com (J.H.); m13525371866@163.com (Y.L.); linqinxiong@mail.scuec.edu.cn (Q.L.); 3 College of Pharmacy, Chengdu University of Traditional Chinese Medicine, Chengdu 611137, China; 4 Guangxi Institute of Medicinal Plant, Nanning 530023, China; chenlulu982@hotmail.com

**Keywords:** Kushenol Z, NSCLC, apoptosis, cAMP, Akt, mTOR

## Abstract

The roots of *Sophora flavescens* (SF) are clinically used as a traditional Chinese medicine for the treatment of various lung diseases. In this study, we investigated the mechanism by which SF inhibits proliferation and induces apoptosis in non-small-cell lung cancer (NSCLC) cells. A new compound, kushenol Z (KZ), and 14 known flavonoids were isolated from SF. KZ, sophoraflavanone G, and kushenol A demonstrated potent cytotoxicity against NSCLC cells in a dose- and time-dependent manner; KZ showed a wide therapeutic window. We also found that KZ induced NSCLC cell apoptosis by increasing the Bax/Bcl-2 ratio and by activating caspase-3 and caspase-9 leading to mitochondrial apoptosis, and upregulated CHOP and activatedcaspase-7 and caspase-12, which triggered the endoplasmic reticulum stress pathway. After KZ treatment, we observed cAMP accumulation, which reflected the inhibition of cAMP-phosphodiesterase (PDE), along with the increase in PKA activity; additionally, phospho-p70 S6 kinase was downregulated. KZ also attenuated the phosphorylation of Akt and PRAS40, which was partially rescued by an Akt activator. This suggested that KZ mediated the antiproliferative activity in NSCLC cells by inhibiting the mTOR pathway through the inhibition of cAMP-PDE and Akt. These findings suggested that KZ may be used as a promising cAMP-PDE and Akt inhibitor in targeted chemotherapeutic drug development.

## 1. Introduction

Lung cancer is a highly prevalent malignancy and the leading cause of cancer-related mortality. Non-small-cell lung cancer (NSCLC) accounts for about 80–85% of lung cancer cases, and the survival rate of patients with advanced NSCLC is comparatively low [1]. Systemic chemotherapy is often the standard treatment option for most patients for whom surgery is not possible. However, serious side effects of chemotherapy has limited treatment success [2,3]. Therefore, it is essential to identify novel compounds with antiproliferative and pro-apoptotic activities that can be used in targeted therapy against NSCLC.

Impaired cyclic nucleotide generation has been observed in various types of cancer. Phosphodiesterases (PDE) is an enzyme that breaks the phosphodiesterase bond and inactivates cyclic nucleotides. PDEs are negative regulators of intracellular cyclic nucleotide generation; dysregulated PDE expression has been observed in lung [4] and brain [5] tumor tissues, and found to be associated with unfavorable clinical prognosis. Notably, previous studies have shown that inhibition of PDEs leads to inhibition of cell proliferation or induction of apoptosis in NSCLC cells [6], glioblastoma stem-like cells [7], and diffuse large B-cell lymphoma cells [8]. These findings suggest that PDEs may be considered as an effective therapeutic target for NSCLC treatment. Similarly, dysregulation of Akt signaling is known to contribute to tumorigenesis. Akt is a classical upstream positive regulator of the mTOR pathway; Akt inhibits PRAS40 and TSC2 to activate mTORC1/2 signaling. Interestingly, studies have demonstrated a crosstalk between cAMP signaling and the Akt/mTOR axis; this may provide a new insight into anticancer effects of PDE inhibitors [8,9]. 

The roots of *Sophora flavescens* (SF) have been officially listed in the *Chinese Pharmacopoeia* and named “Ku Shen”; SF has been used in the treatment of various malignancies, including liver and lung cancer. Our previous screening of pre-fractionated extract from SF displayed strong cytotoxic activity against NSCLC cells. An array of reports also reached a similar consensus that SF possesses anticancer activity against NSCLC [10,11,12,13,14,15]. Therefore, we aimed to identify a novel compound derived from a traditional Chinese medicine (TCM), and investigated the underlying mechanism by which it mediated antiproliferative and pro-apoptotic effects in NSCLC cells. In this study, a new flavonoid named kushenol Z (KZ) and 14 known flavonoids were isolated from SF using the bioassay-guided separation technique (Supplementary Figure S1). Herein, KZ is shown to exhibit potent antineoplastic property against NSCLC cells. Furthermore, an interesting finding that KZ treatment increased cAMP level and inhibited Akt activity motivated us to investigate whether KZ is a potential inhibitor of cAMP-PDE and Akt is implicated in the inhibition of NSCLC. Further studies were thus designed to explore the possible molecular mechanisms underlying the anti-NSCLC activity of KZ.

## 2. Results

### 2.1. Structure of KZ

**Figure 1:**
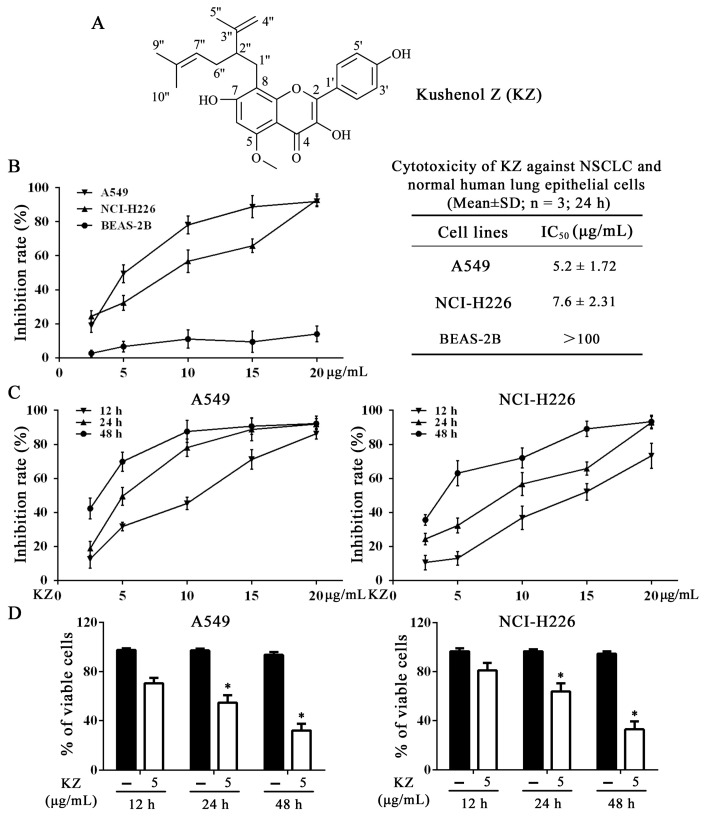
Kushenol Z (KZ) inhibits non-small-cell lung cancer (NSCLC) cell proliferation. (**A**) Structures of KZ. (**B**) Cytotoxicity was determined using CCK-8 assay. NSCLC cell lines, A549 and NCI-H226, and normal human lung epithelial cell line, BEAS-2B, were treated with indicated concentrations of KZ for 24 h. (**C**) A549 and NCI-H226 cells were treated with indicated concentrations of KZ for 12, 24, or 48 h. (**D**) Cell viability was determined using trypan-blue exclusion assay. A549 and NCI-H226 cells were treated with KZ (5 μg/mL) for 12, 24, or 48 h. The data are shown as the means ± SD of three independent experiments. * *p* < 0.05 compared with the 12 h group.

Compound **1** appeared as a yellow powder. The chemical formula of compound **1** was C_26_H_28_O_6_ and was derived from HR-ESI-MS data ([M + Na]^+^ 459.1772, calcd. 459.1784). The UV spectrum showed absorption maxima at 269, 308, and 363 nm. The ^1^H-nuclear magnetic resonance spectroscopy (NMR) signals (Supplementary Table S1) showed a typical 1, 4-disubstituted aromatic proton at *δ*_H_ 8.04 (2H, d, *J* = 8.9 Hz, H-2′, 6′), *δ*_H_ 6.91 (2H, d, *J* = 8.9 Hz, H-3′, 5′), and an isolated aromatic proton at *δ*_H_ 6.44 (1H, s, H-6), which were characteristic of flavonol derivatives [16]. Additional signals at *δ*_H_ 4.96 (1H, t, *J* = 6.8 Hz, H-7”), 4.48 (1H, s, H-4′′α), 4.62 (1H, s, H-4′′β), 2.86 (2H, m, H-1′′), 2.52 (1H, m, H-2′′), 2.08 (2H, t, *J* = 6.8 Hz, H-6”), 1.67 (3H, s, H-5′′), 1.56 (3H, s, H-9”), and 1.48 (3H, s, H-10”) were attributed to a lavandulyl group [16]. Furthermore, the HMBC correlation (Supplementary Figure S2) of the proton at *δ*_H_ 2.86 (H-1′′) with the aromatic carbon at *δ*_C_ 106.5 (C-8), 160.6 (C-7), and 156.2 (C-8a) indicated that the lavandulyl group was attached at C-8, suggesting a chemical structure similar to the known flavonoid kushenol C [16]. Compared with kushenol C, the spectrum of compound **1** showed an additional methoxy group at C-5 and a hydrogen atom at C-2′ instead of a hydroxyl group. The HMBC correlation between the methoxy group at *δ*_H_ 3.81 (-OCH_3_) and the aromatic carbon at *δ*_C_ 158.4 (C-5) indicated that the methoxy group was attached to C-5. Furthermore, the ^13^C-NMR spectrum of compound **1** showed signals from four oxygenated aromatic carbons. Chemical shifts and signal patterns in ^1^H-NMR and ^13^C-NMR (Supplementary Table S1) indicated that the hydroxyl groups were at C-3, C-7, and C-4′. Thus, compound **1** was identified as 8-(2-isopropenyl-5-methylhexyl)-5-methoxy-3,7,4′-trihydroxyflavone (Figure 1A), and was named kushenol Z in accordance with trivial names of the related lavandulyl flavonoids from SF. 

Based on the 1D and 2D NMR and mass spectrometry data, we identified 14 additional known compounds (Supplementary Figure S1), namely trifolrhizin (compound **2**) [17], calycosin (**3**) [18], desmethylanhydroicaritin (compound **4**) [19], sophoflavescenol (compound **5**) [19], (*2R*)-3α,7,4’-trihydroxy-5-methoxy-8-(γ,γ-dimethylallyl)-flavanone (compound **6**) [20], (*2S*)-7,2’,4’-trihydroxy-5-methoxy-8-dimethylallyl flavanone (compound **7**) [15], 8-dimethylallyltsugafolin (compound **8**) [21], kushenol N (compound **9**) [22], sophoraflavanone G (compound **10**) [23], leachianone A (compound **11**) [24], xanthohumol (compound **12**) [25], kuraridin (compound **13**) [23], kushenol D (compound **14**) [24], and kushenol A (compound **15**) [26]. 

### 2.2. KZ Inhibits NSCLC Cell Proliferation

SF extracts have been reported to demonstrate anticancer activity against different malignancies including hepatocellular carcinoma and NSCLC [10]. Therefore, we evaluated the cytotoxic activity of the isolated compounds using MTT and trypan-blue exclusion assays in NSCLC cell lines (A549 and NCI-H226) and the human bronchial epithelial cell line (BEAS-2B). As shown in Supplementary Table S2 and Figure S3, KZ, compound **10**, and compound **15** were the most potent inhibitors of NSCLC cell proliferation; in contrast, compound 10 and compound 15 induced stronger inhibition of BEAS-2B cell proliferation than KZ. Using cell counting kit-8 (CCK-8) assay, we found that KZ selectively inhibited the proliferation of NSCLC cell and not normal lung cells (Figure 1B). This suggested that a potential role of KZ in NSCLC treatment. The inhibition curve and trypan-blue exclusion assay further showed that KZ inhibited NSCLC cell proliferation in a dose- and time-dependent manner (Figure 1D). Notably, these data showed that A549 cells were more sensitive to KZ-mediated inhibition of cell proliferation. Taken together, KZ exhibited anti-proliferative activity selectively against NSCLC in a dose- and time-dependent manner.

### 2.3. KZ Induces NSCLC Cell Apoptosis

**Figure 2:**
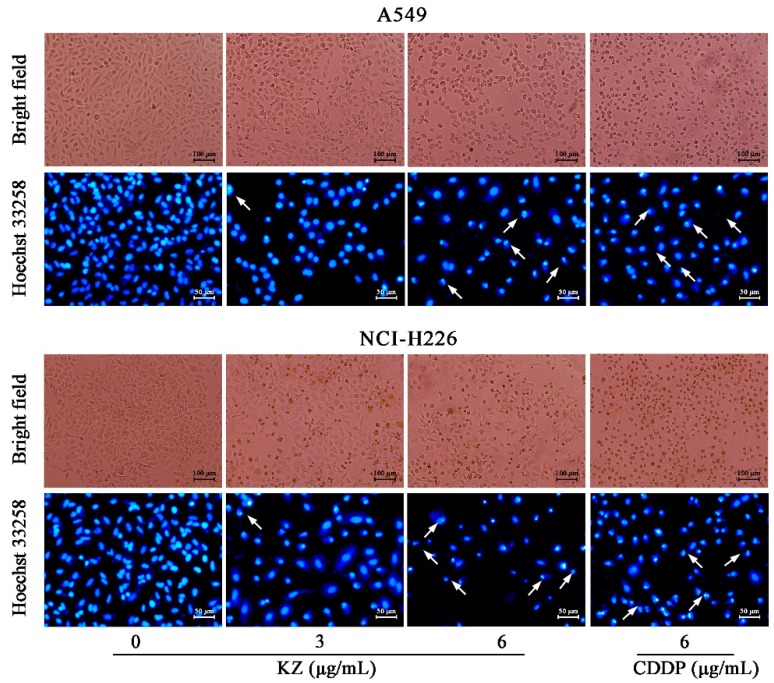
KZ induced morphological changes in NSCLC cells indicating apoptosis. A549 and NCI-H226 cells were treated with indicated concentrations of KZ or CDDP for 24 h. Cisplatin (CDDP) served as the positive control. Bright field images were acquired using a phase contrast microscope. Thereafter, the cells were fixed and stained by Hoechst 33258. Hoechst 33258 stained images were acquired using a fluorescence microscope. Chromatin condensation and apoptotic body formation are indicated by arrowheads.

**Figure 3:**
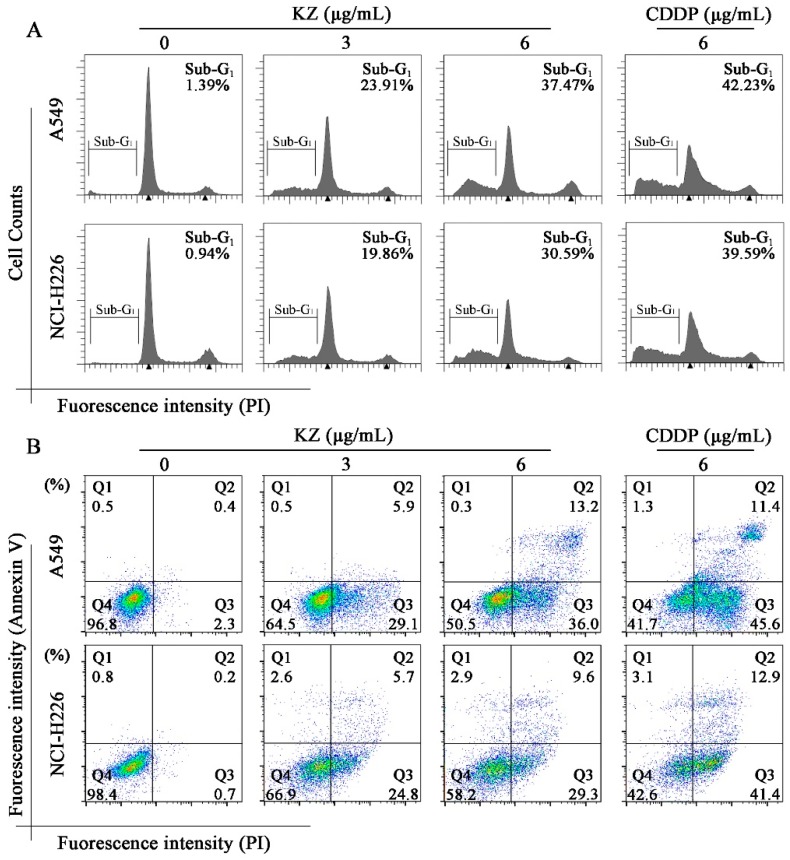
Flow cytometric analysis for determining KZ-induced NSCLC cell apoptosis. A549 and NCI-H226 cells were treated with indicated concentrations of KZ or CDDP for 24 h. CDDP was used as the positive control. (**A**) Propidium iodide (PI) staining was performed to detect the sub-G_1_ peak. (**B**) Annexin V/PI staining was used to monitor the apoptotic progress. Q4 indicated non-apoptotic cells; Q3 and Q2 indicated the viable and non-viable stages of apoptosis, respectively; Q1 indicated necrocytotic cells.

To explore whether KZ triggered NSCLC cell apoptosis, morphological and flow cytometric analysis (FACS) were performed. The pro-apoptotic effect of cisplatin (CDDP) is well known, and hence, CDDP was used as the positive control. We observed that progressive KZ treatment induced morphological changes in NSCLC cells such as shrinkage, distortion, and floatage. Hoechst 33258 nuclear staining revealed that KZ treatment induced chromatin condensation and apoptotic body formation similar to that triggered by CDDP (Figure 2). Sub-G_1_ peak is a typical apoptotic signal in single nuclear staining because apoptotic cell’s chromosome degradation generally results in the fluorescence decrease compared with the normal cell. Moreover, classic features of apoptotic cells including phosphatidylserine exposure and increased membrane permeability can be investigated using annexin V and propidium iodide staining (PI), respectively, which helps to distinguish between apoptosis and necrocytosis. FACS of PI stained A549 and NCI-H226 cells showed that KZ treatment increased the percentage of cells in the sub-G_1_ peak in a dose-dependent manner (Figure 3A). FACS of annexin V/PI staining further confirmed that KZ treatment increased apoptosis (Q2 and Q3, Figure 3B). These data suggested that KZ mediates anti-cancer effect by inducing apoptosis of NSCLC cells.

### 2.4. KZ Promotes NSCLC Cell Apoptosis by Mitochondrial and Endoplasmic Reticulum Stress Pathways

**Figure 4:**
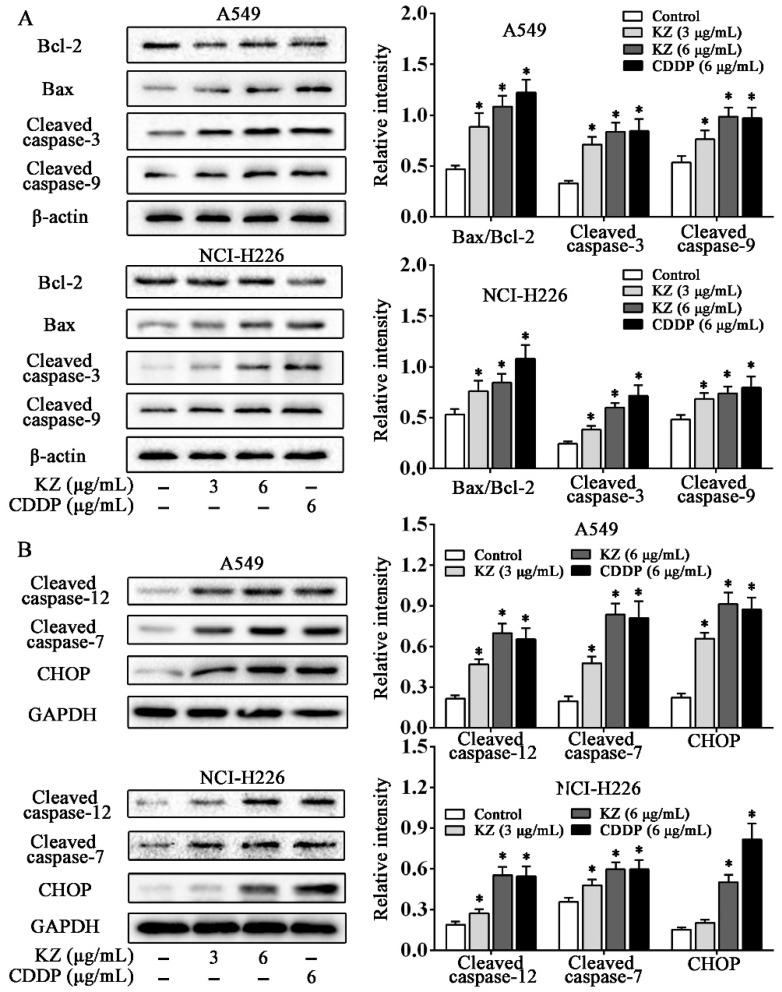
KZ promotes NSCLC cell apoptosis through the mitochondrial and endoplasmic reticulum stress pathways. A549 and NCI-H226 cells were treated with indicated concentrations of KZ or CDDP for 24 h; thereafter, whole-cell lysates were processed for Western blot analysis and probed with the indicated antibodies. (**A**) Expression proteins involved in the mitochondrial apoptotic pathway: Bcl-2, Bax, cleaved caspase-3, and caspase-9. (**B**) Expression proteins involved in the endoplasmic reticulum stress pathway: Cleaved caspase-12, caspase-7, and CHOP. Relative intensity is shown as the means ± SD of three independent experiments. * *p* < 0.05 compared with control.

We used several apoptotic markers to investigate the underlying mechanisms of KZ-induced apoptosis. In mitochondrial pathway of apoptosis, the change in the Bax/Bcl-2 ratio initiates the caspase cascade [27]. Western blot analysis showed that KZ upregulated the ratio of Bax/Bcl-2, leading to the cleavage of caspase-9 and caspase-3 in A549 and NCI-H226 cells (Figure 4A). In the endoplasmic reticulum stress (ERS) pathway, the expression of the ERS marker, CHOP is upregulated. We observed that KZ treatment resulted in an increased cleavage of caspase-7 and caspase-12 to a greater degree than CDDP in A549; a similar effect was observed in CHOP expression. These data revealed that mitochondrial and endoplasmic reticulum stress pathways are involved in KZ-mediated NSCLC cell apoptosis. 

### 2.5. KZ Mediates Anti-NSCLC Effects by Inhibiting the mTOR Pathway

#### 2.5.1. KZ Upregulates cAMP Levels to Increase PKA Activity That Causes Inhibition of mTORC1 

**Figure 5:**
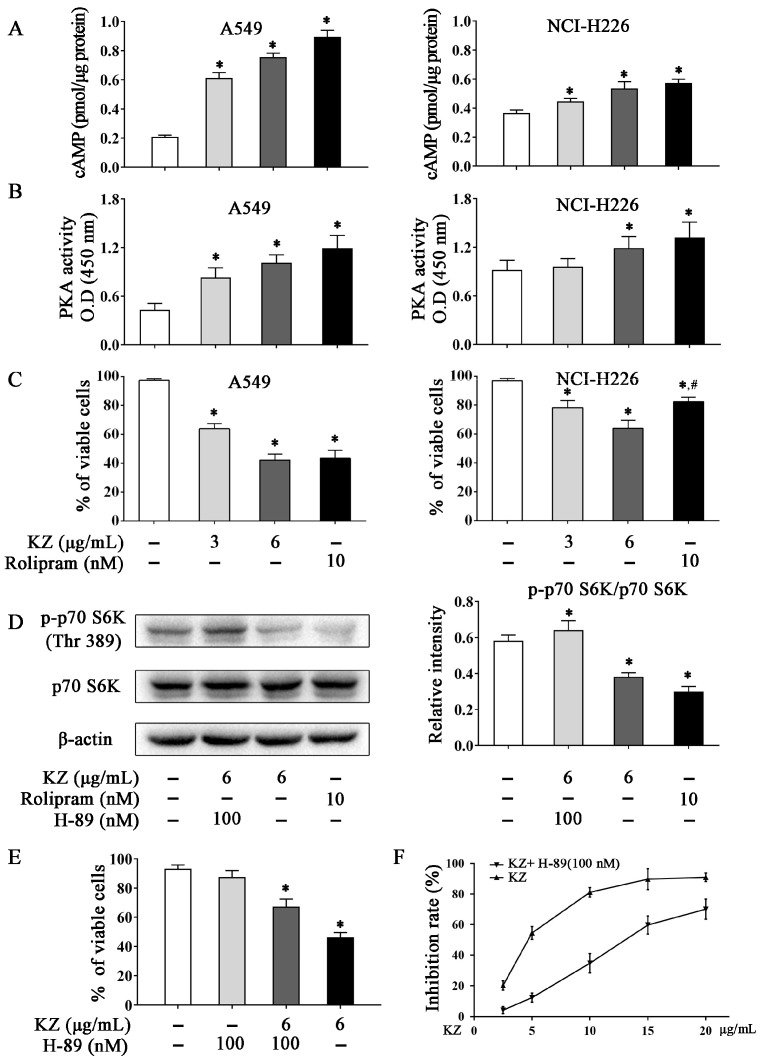
KZ induced cAMP-dependent mTORC1 inhibition by increasing PKA activity. A549 and NCI-H226 cells were treated with indicated concentrations of KZ, rolipram, or H-89 for 24 h. Rolipram and H-89 served as the positive and negative controls, respectively. (**A**) Inhibition of cAMP-phosphodiesterase (PDE) by KZ and rolipram. Intracellular cAMP levels were determined using cAMP enzyme immunoassay. (**B**) PKA activity was determined using the PKA kinase activity assay kit; absorbance was measured at 405 nm. (**C**) Anti-proliferative effect of KZ and rolipram was determined using trypan-blue exclusion assay. (**D**) Protein levels of p-p70 S6K (Thr 389) and p70 S6K in A549 cells were determined by Western blotting. (**E, F**) Effect of KZ and H-89 on proliferation. Cell viability was determined using trypan-blue exclusion assay. Cytotoxicity was determined using CCK-8 assay. The data are shown as the means ± SD of three independent experiments. * *p* < 0.05 compared with the untreated group, ^#^ *p* < 0.05 compared with the KZ-treated (6 μg/mL) group.

Several flavonoids with a structure similar to KZ have been reported to inhibit cAMP-PDE [28]. Recent studies have implicated a role of dysregulated PDEs in the metastasis of lung cancer, abnormal cell proliferation, and apoptosis resistance [29]. Hence, inhibition of PDEs is a promising therapeutic option for lung cancer. To investigate whether KZ is a potential inhibitor of cAMP-PDE, we detected the cAMP level in A549 and NCI-H226 cells, and used rolipram as the positive control. We found that KZ and rolipram treatment increased the intracellular concentration of cAMP in A549 cells, while the elevation of cAMP levels in NCI-H226 cells was intermediate (Figure 5A). Similarly, KZ treatment increased the activity of PKA, a major effector of cAMP (Figure 5B). Trypan blue exclusion assay revealed that KZ inhibited both A549 and NCI-H226 cell proliferation, as described previously (Figure 5C). Notably, rolipram showed a similar effect in A549, whereas NCI-H226 cells were insensitive to the antiproliferative effect of rolipram (Figure 5C). These results suggested that KZ inhibited proliferation by inhibiting cAMP-PDE in A549, and the sensitivity in response to KZ treatment may correlate with the intrinsic cAMP-PDE activity. Recent studies showed that cAMP-dependent inhibition of the mTOR pathway is mediated via PKA [9]. To evaluate whether PKA is involved in mediating the anti-proliferative effect of KZ, we used the PKA selective inhibitor H-89 in combination with KZ in KZ-sensitive A549 cells. We found that KZ and rolipram decreased the phosphorylation of p70 S6 kinase (p-p70 S6K), which was reversed by H-89 treatment (Figure 5D). Proliferation assay further showed that the KZ-induced inhibition of cell proliferation could be partially rescued by H-89 (Figure 5E,F). Taken together, these results indicated that KZ is a potential inhibitor of cAMP-PDE that leads to increased PKA activity and inhibition of the mTOR pathway. 

#### 2.5.2. KZ-Induced Antiproliferative Effect Involves the Inhibition of Akt

**Figure 6:**
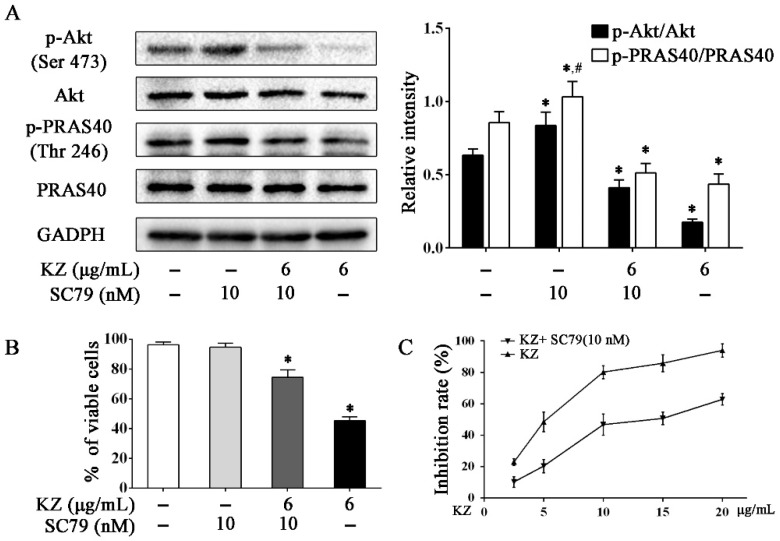
KZ inhibited the mTOR pathway by inhibiting Akt. A549 cells were treated with indicated concentrations of KZ or SC79 for 24 h. SC79, an Akt activator, served as the negative control. (**A**) Protein level of p-Akt (Ser 473), Akt, p-PRAS40 (Thr 246), and PRAS40 were determined by Western blotting. (**B, C**) Effect of KZ and SC79 on proliferation. Cell viability was determined using trypan-blue exclusion assay. Cytotoxicity was determined using CCK-8 assay. The data are shown as the means ± SD of three independent experiments. * *p* < 0.05 compared with the untreated group, ^#^ *p* < 0.05 compared with the KZ-treated (6 μg/mL) group.

To further understand the mechanism by which KZ inhibits cell proliferation, Western blotting was performed to detect Akt activity in A549 cells. Interestingly, data showed that KZ treatment downregulated the phosphorylation of Akt, accompanied by decreased phosphorylation of PRAS40, which is an upstream inhibitor of mTOR. PRAS40 is known to be inactivated by Akt. Akt activator, SC79 reversed KZ-mediated inhibition of phosphorylation of Akt (Figure 6A). Proliferation assay showed that the KZ-induced inhibition of cell proliferation could be partially rescued by SC79 (Figure 6B,C). These results indicate that KZ also mediates antiproliferative effects by inhibiting Akt activity that leads to the inhibition of the mTOR pathway.

## 3. Discussion

The dried roots of SF are known as Kushen in TCM, and was first described in 200 A.D. as a therapeutic option for solid tumors, inflammation, and other diseases [10]. Kushen alkaloids and kushen flavonoids are the bioactive components of SF. In this study, 15 flavonoids were isolated from the ethyl acetate extract of the roots of SF; among these, KZ was identified as a new compound. We investigated the anti-proliferative activities of these compounds, in NSCLC cells and normal human lung cells. KZ, compound **10**, and compound **15** were shown to exhibit considerable cytotoxic effects against NSCLC cells. These data were consistent with previous studies in which compound **10** and compound **15** demonstrated broad-spectrum anti-tumor activity [10,13]. 

Platinum-based regimens plus gemcitabine, although widely recommended for the treatment of advance lung cancer, has several side effects including acute/sub-acute toxicity and myelosuppression; this often results in poor tumor response, substandard quality of life, and poor prognosis [30,31]. Therefore, alternative, well-tolerated treatment options are urgently required. We noted that compound **10** and compound **15** were distinctly cytotoxic to the untransformed pneumonocyte. Here, we showed that KZ exhibits NSCLC-selective cytotoxic and inhibited NSCLC cells proliferation in a dose- and time-dependent manner, suggesting the potential anti-cancer property of KZ against NSCLC. 

Apoptosis is a self-destruction program to remove cells that are in excess or potentially dangerous. Accordingly, it is recognized that pro-apoptotic strategy plays a crucial role in the chemotherapy against NSCLC. Notably, KZ treatment resulted in morphological changes in NSCLC cells that were indicative of apoptosis. By using PI and annexin V/PI staining that specifically indicated early apoptosis to late apoptosis, we demonstrated the potent pro-apoptotic effects of KZ on NSCLC cells. Mechanistically, we showed that KZ synergistically promoted apoptosis through the mitochondrial pathway, by regulating the Bax/Bcl-2 ratio and activating of caspase-3 and caspase-9, and through the endoplasmic reticulum stress pathway by activating caspase-12 and caspase-7 and by upregulating CHOP.

Although apoptosis is generally conserved in many cancers, it is showed clearly that the dysregulation of upstream survival pathway significantly inhibits this process for adaptation to cell stress. Therefore, it is conceivable that targeting these signals may sensitize tumor cells to apoptosis. PDEs are a class of enzymes that are involved in many pathophysiological processes including cell proliferation and differentiation, cell-cycle regulation, gene expression, inflammation, apoptosis, and metabolic functions [32]. Dysregulation of PDEs is correlated with carcinogenesis because overexpression of PDEs has been observed in various malignancies [33,34], including lung cancer [4]; dysregulation of PDEs in cancer cells results in lower level of cAMP/cGMP than that in normal cells. Sufficient evidence has been provided regarding the key role of PDE inhibitors, such as rolipram, theophylline, or aminophylline, in the regulation of cell proliferation and treatment of cancer [29,35]. A preliminary study revealed the potential role of KZ in the inhibition of cAMP-PDE [28]. Our study showed that KZ increases the intracellular cAMP concentration, accompanied by an increase in PKA activity in NSCLC cells; this indicated the inhibitory effect of KZ on cAMP-PDE. Recent studies showed that PKA is also involved in the inhibition of the mTOR pathway [9]. We found that KZ and rolipram treatment decreased the phosphorylation of p70 S6K at Thr389 in A549 cells, which was reversed by treatment with a PKA inhibitor (H-89); this suggested that KZ upregulated cAMP levels leading to an increase in PKA activity and inhibition of mTORC1, which in turn, results in inhibition of cell proliferation. It was reported that the H-89 also inhibited p70 S6K [36], which might contribute to the unremarkable antiproliferation treated with H-89 independently. Furthermore, the selective inhibition of rolipram between A549 and NCI-H226 that was observed, except for KZ, suggested the possibility that KZ is a non-selective inhibitor against PDE4 or there exist more mechanisms that underlie KZ-induced antiproliferation. Interestingly, we further found that KZ inhibited the phosphorylation of Akt, which is a classical upstream positive regulator of the mTOR pathway. Akt inhibits PRAS40, which is an inhibitor of mTORC1. Combined treatment with KZ and Akt activator reversed the inhibitory effect of KZ on phosphorylation of Akt and PRAS40, and eventually rescued A549 cells, suggesting that Akt is also a potential target of KZ. These results revealed that KZ inhibited the mTOR pathway by activating PKA and inhibiting Akt activities to induce apoptosis in NSCLC cells.

**Figure 7:**
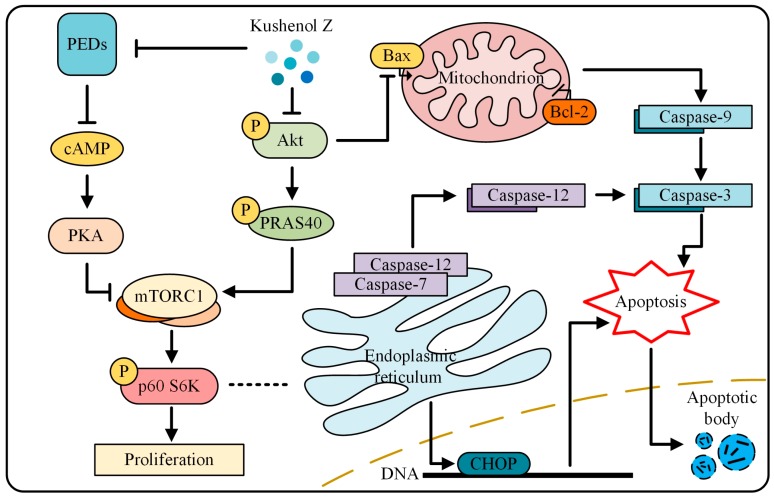
The mechanism of KZ in NSCLC cells. KZ inhibited cAMP-PDE that resulted in the accumulation of cAMP leading to an increased PKA activity. Additionally, KZ inhibited Akt activity leading to a reduction in the inhibition of PRAS40, and thus impedes proliferation leading to mitochondria and endoplasmic-reticulum apoptosis.

In summary, we isolated from SF a novel flavonoid called KZ that demonstrated potent antiproliferative and pro-apoptotic activities in NSCLC cells; KZ showed only moderate cytotoxicity in normal lung epithelial cells. Mechanistically, our study revealed that KZ inhibited cAMP-PDE that resulted in the accumulation of cAMP leading to an increased PKA activity. Additionally, KZ inhibited Akt activity leading to a reduction in the inhibition of PRAS40, and thus impedes proliferation leading to mitochondria and endoplasmic-reticulum apoptosis. These findings are outlined in Figure 7 and suggest that KZ may be used as a potential cAMP-PDE and Akt inhibitor in targeted chemotherapeutic drug development.

## 4. Materials and Methods 

### 4.1. Plant Materials

The roots of *Sophora flavescens* (SF) were collected from Lingyuan City, Liaoning province, China in Sept, 2018, and identified by Professor Dingrong Wan of School of Pharmaceutical Sciences, South-Central University for Nationalities, Wuhan, China. A voucher specimen (No. SC0060) was deposited in School of Pharmaceutical Sciences, SCUN, Wuhan, China.

### 4.2. Extraction and Isolation

General experimental procedures and detailed spectroscopic data of isolation from SF are shown in Supplementary Material. In brief, air-dried roots of SF (500 g) were triturated and then extracted sequentially by maceration with n-hexane (4 × 2.0 L, 5 h each) at room temperature, followed by ethyl acetate (4 × 2.0 L, 5 h each) and methanol (4 × 2.0 L, 5 h each). The solvents were evaporated at reduced pressure to yield 4.9 g, 36.8 g, and 58.7 g of n-hexane, ethyl acetate (SF-EtOAc) and methanol fractions, respectively. A new compound kushenol Z and 14 known flavanoids were isolated from the EtOAc extract (23 g) by spectroscopic and chemical methods.

### 4.3. Kushenol Z (1, KZ)

Light yellow powder; UV (MeOH) λ_max_ (log ε): 269, 308, 363. IR (film) ν_max_: 3300 (br), 1610, 1564, 1268. ^1^H- and ^13^C-NMR data are described in Supplementary Material. HRESIMS *m/z* 459.1772 (calcd. for C_26_H_27_O_6_Na^+^, 459.1784).

### 4.4. Cell Culture and Reagents

The NSCLC cell line A549 and NCI-H226 and the human bronchial epithelial BEAS-2B cell lines were purchased from the American Type Culture Collection (ATCC; Manassas, VA, USA). The A549, NCI-H1975 and BEAS-2B cells were grown in a DMEM medium (Gibco, Grand Island, NY, USA) supplemented with 10% fetal bovine serum (Gibco, Grand Island, NY, USA) and 1% penicillin/streptomycin in a humidified atmosphere containing 5% CO_2_ at 37 °C.

### 4.5. CCK-8 Assay

Cells (1 × 10^4^ cells/well) were seeded into 96-well plates and were treated indicated condition. After the indicated time, supernate was abandoned and 100 μL of CCK-8 (5 mg/mL) dissolved in medium was added to each well and incubated for 2 h. The absorbance was measured at 450 nm by a Microplate Reader (BIO-RAD, Hercules, CA, USA).

### 4.6. Trypan-Blue Exclusion Assay

Trypan-blue exclusion assay was performed as described previously [37]. Cells (1 × 10^5^ cells/well) were seeded into 6-well plates, treated with indicated condition. Following the treatment time, cells were then collected and stained with trypan blue (Beyotime, Shanghai, China). The viable and dead cells (stained by trypan blue) were counted by a hematocytometer. The percentage of viable cells was plotted graphically with histogram for quantification of cell viability.

### 4.7. Observation of Morphological Changes

Cells (1 × 10^5^ cells/well) were seeded into 6-well plates, treated with indicated condition. Subsequently, a phase contrast microscope (Leica, Nussloch, Germany) was used to observe the cellular morphological changes.

### 4.8. Hoechst 33258 Staining Assay

Hoechst 33258 staining assay was performed as described previously [38]. Cells (1 × 10^5^ cells/well) were seeded into 6-well plates for 24 h. Then, the cells and were treated with indicated condition. After discarding the supernatant, 1.0 mL of stationary liquid (methanol: acetic acid = 3:1) were covered for about 30 min. Then, Hoechst 33258 solution (5 μg/mL) was added to the wells for 30 min. The cells were observed under a fluorescence microscope (Leica Microsystems, Wetzlar, Germany).

### 4.9. Flow Cytometry Analysis (FACS)

Cells (1 × 10^5^–4 × 10^5^ cells/well) were seeded in 6-well plates and treated with indicated condition. Then, the cells were collected, fixed in 70% ethanol at 4 °C overnight, washed in PBS, and stained by 100 μL RNase A and 400 μL PI. The cell cycle distribution analysis was measured using a flow cytometer (BD Biosciences, Franklin Lakes, NJ, USA).

### 4.10. Western Blot Analysis and Immunoassay

Cells (1 × 10^5^–4 × 10^5^ cells/well) were seeded in 6-well plates and treated with indicated condition. Then, the cells were lysed and incubated on ice for 30 min. After being centrifuged at 12,000 rpm for 15 min, the protein was separated by electrophoresis on 12% SDS-PAGE and transferred to polyvinylidine difluoride (PVDF) membrane (Bio-Rad, Hercules, CA, USA). Membranes were blocked in 5% skimmed milk and incubated with indicated primary antibodies. The incubated mixture was washed with TBST. Then horseradish peroxidase (HRP) secondary antibodies were added with the mixture incubating at 37 °C for 2 h. The incubated mixture was washed with TBST. HRP electrogenerated chemiluminescence (ECL) was used to develop, the developed films were taken and rinsed with pure water, and the washed films were dried. Scanning was used for recording.

cAMP levels were measured by the cAMP Direct Immunoassay Kit (Abcam, Cambridge, MA, USA) following Abcam’s protocol. PKA activity was evaluated by the PKA Kinase Activity Assay Kit (Abcam, Cambridge, MA, USA) and following Abcam’s protocol. Briefly, after treatment, the protein content of each sample was determined with Bradford assay; 10 µg of each lysates was used for detecting optical density at 450 nm by ELISA method.

### 4.11. Statistical Analysis

All data were expressed as mean ± SD from three independent experiments. One-way analysis of variance (ANOVA) was used for multiple group comparisons using GraphPad Prism 5.0 software package. *p*-values < 0.05 were considered significant.

## Supplementary Materials

The following are available online at https://www.mdpi.com/1420-3049/24/24/4425/s1, Figure S1: Structures of compounds **1**–**15**, Figure S2: Key HMBC (→) of compound **1**, Figure S3: Inhibitory effect of SF isolation on NSCLC cell proliferation, Table S1: ^1^H and ^13^C NMR data of compound **1** (δ in ppm, *J* in Hz), Table S2: The half maximal inhibitory concentration values (IC_50_, μg/mL) of the tested 15 compounds for NSCLC cell lines (A549 and NCI-H226) and human bronchial epithelial cell lines (BEAS-2B).
