# Supplementary material for: A Novel Flavonoid Kushenol Z from Sophora flavescens Mediates mTOR Pathway by Inhibiting Phosphodiesterase and Akt Activity to Induce Apoptosis in Non-Small-Cell Lung Cancer Cells
Source: Molecules. 2019 Dec 4;24(24):4425. doi: 10.3390/molecules24244425 (PMC6943755; doi:10.3390/molecules24244425)
Supplement: Supplementary file 1 [file molecules-24-04425-s001.pdf]

Supplementary Material

1. Materials and Methods

1.1. General Experimental Procedures

UV and IR spectra were recorded on a Hewlett-Packard 8452A diode array spectrophotometer (Agilent, Lexington, MA, USA) and a Nicolet Magna FT-IR 750 spectrometer (Nicolet, Mountain, WI, USA), respectively. LC-PDA-ESIMS data were recorded on a Waters ACQUITY SQD MS system (Waters, Milford, MA, USA) connected to a Waters 1525 HPLC with a 2998 Photodiode Array Detector (Waters, Milford, MA, USA) and a Waters SunfireTM C18 column (5 μm, 4.6 × 150 mm). NMR (MeOH-d4 or DMSO-d6) spectra were acquired on an AVANCE III 600 MHz NMR spectrometer equipped with Micro NMR tubes (1.4 mm). The chemical shifts (δ) were reported in ppm, and coupling constants (*J*) were given in Hz. The ESIMS and HRESIMS data were recorded on a Q-TOF Micro LC-MS-MS mass spectrometer. A Thermo C18 5 μm column (22 mm × 150 mm) was used for semi-preparative HPLC. A Waters 2535 HPLC fitted with a 2998 Photodiode Array Detector and a 2707 Autosampler was used for the semi-preparative separations. Silica gel GF254 precoated glass plates (1.00 mm, Yantai Jiangyou Silica Development Co., Ltd, Yantai, China) were used for preparative TLC (PTLC). Sephadex LH-20 (Amersham Pharmacia Biotech Co., Amersham, UK) was also used for column chromatography.

1.2. MTT Assay

Cell viability of A549, NCI-H226, and BEAS-2B were analyzed using 3-(4,5-dimethylthiazol-2-yl)-2,5-diphenyltetrazolium bromide (MTT) assay [1]. Cells were seeded at 1×10^4 cells/well into 96-well plates and were treated with 16 compounds at the indicated condition. After that, 100 uL of MTT solution dissolved in medium was added to each well and incubated for 2 h. Then, DMSO was used to dissolve the formazan crystals and the absorbance was measured at 492 nm by a Microplate Reader (BIO-RAD).

1.3. Trypan-Blue Exclusion Assay

Cells (1×10^5 cells/well) were seeded into 6-well plates and treated with indicated condition. Following the treatment time, cells were then collected and stained with trypan blue (Beyotime, Shanghai, China). The viable and dead cells (stained by trypan blue) were counted by a hematocytometer. The percentage of viable cells was plotted graphically with histogram for quantification of cell viability.

2. Supplementary Figures and Legends

2.1. Chemical Structures of Compounds **1–15**

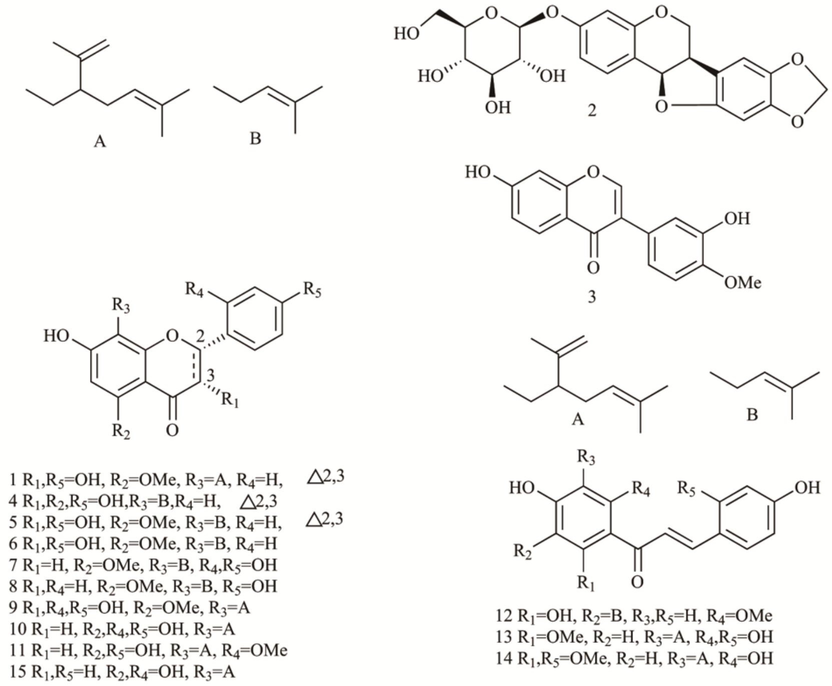

Supplementary Figure 1. Structures of compounds **1–15**.

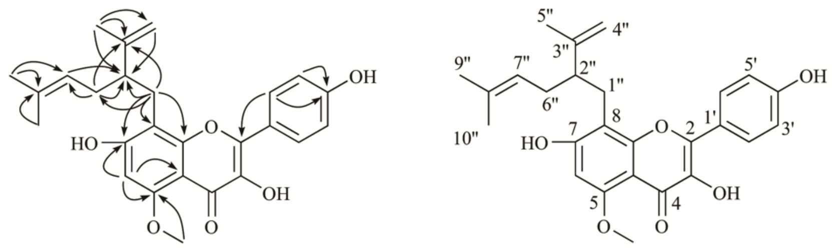

Supplementary Figure 2. Key HMBC (→) of compound **1**.

Supplementary Table 1. ^{1}H and ^{13}C NMR data of compound 1 (δ in ppm, *J* in Hz).

No.	δ_H ^{a,b} (*J* in Hz)	δ_C ^{a,c}	HMBC (H→C)
2		142.1	
3		137.3	
4		171.7	
4a		105.5	
5		158.4	
6	6.44 (s)	95.6	4, 4a, 5, 7
7		160.6	
8		106.5	
8a		156.2	
1'		122.7	
2'	8.03 (d, 8.9)	129.1	2, 3', 4'
3'	6.91 (d, 8.9)	115.8	1', 2', 4'
4'		158.9	
5'	6.91 (d, 8.9)	115.8	

6'	8.03 (d, 8.9)	129.1	
1''	2.86 (m)	27.6	7, 8, 8a, 2'', 3'', 6''
2''	2.52 (m)	47.3	8, 1'', 3'', 4'', 5'', 6'', 7''
3''		147.9	
4''	4.48 (s), 4.62 (s)	111.7	2'', 3'', 5''
5''	1.67 (s)	18.8	2'', 3'', 4''
6''	2.08 (t, 6.8)	31.2	1'', 2'', 3'', 7'', 8''
7''	4.96 (t, 6.8)	123.4	2'', 6'', 9'', 10''
8''		131.4	
9''	1.47 (s)	18.1	7'', 8''
10''	1.56 (s)	26.0	7'', 8''
$-OCH_3$	3.81 (s)	56.1	5

[a] Measured in DMSO-d_6; [b] at 600 MHz; [c] at 150 MHz.

2.2. KZ, Compounds **10,** *and* **15** *Inhibit Growth of NSCLC Cell Lines*

All isolated compounds were primarily tested for their anti-tumor activity against NSCLC cell lines (A549 and NCI-H226) and normal human lung epithelial cells (BEAS-2B) using MTT assay and trypan-blue exclusion assay. As presented in Supplementary Table 2, KZ, **10,** and **15** were potent to inhibit the proliferation of NSCLC cells, while KZ showed more moderate toxic effects on BEAS-2B (IC_{50} > 50 μg/mL). Trypan-blue exclusion assay further confirmed that KZ exhibited a promising inhibitory effect on NSCLC cells with an acceptable therapeutic window compared with **10** and **15** (Supplementary Figure 2).

Supplementary Table 2. The half maximal inhibitory concentration values (IC_{50}, μg/mL) of the tested 15 compounds for NSCLC cell lines (A549 and NCI-H226) and human bronchial epithelial cell lines (BEAS-2B).

No.	IC_{50} (μg/mL) ± SD		
	A549	NCI-H226	BEAS-2B
**1**	4.8 ± 4.3	11.3 ± 5.5	126.9 ± 8.6
**2**	147.3 ± 10.8	184.4 ± 13.2	189.4 ± 12.3
**3**	104.2 ± 7.7	97.3 ±6.0	152.6 ± 8.9
**4**	67.8 ± 7.2	104.2 ± 9.5	141.2 ± 10.2
**5**	74.3 ± 4.4	96.2 ± 8.1	173.3 ± 7.6
**6**	57.8 ± 5.3	62.4 ± 7.3	154.6 ± 6.2
**7**	72.3 ± 5.9	87.8 ± 4.3	103.4 ± 7.1
**8**	40.8 ± 3.8	47.7 ± 3.7	104.7 ± 6.8
**9**	21.3 ± 1.8	28.8 ± 1.6	81.6 ± 7.3
**10**	5.8 ± 0.9	16.4± 2.3	51.6 ± 4.2

**11**	25.2 ± 2.4	36.4 ± 4.7	96.4 ± 6.2
**12**	33.5 ± 2.9	41.2 ± 3.4	76.3 ± 8.6
**13**	22.8 ± 1.3	26.3 ± 1.9	74.1 ± 5.5
**14**	21.3 ± 1.5	25.8 ± 1.6	89.1 ± 7.2
**15**	5.3 ± 1.3	20.5 ± 3.7	57.2 ± 5.1
**CDDP**	23.2 ± 1.8	33.2 ± 2.8	22.5 ± 2.7

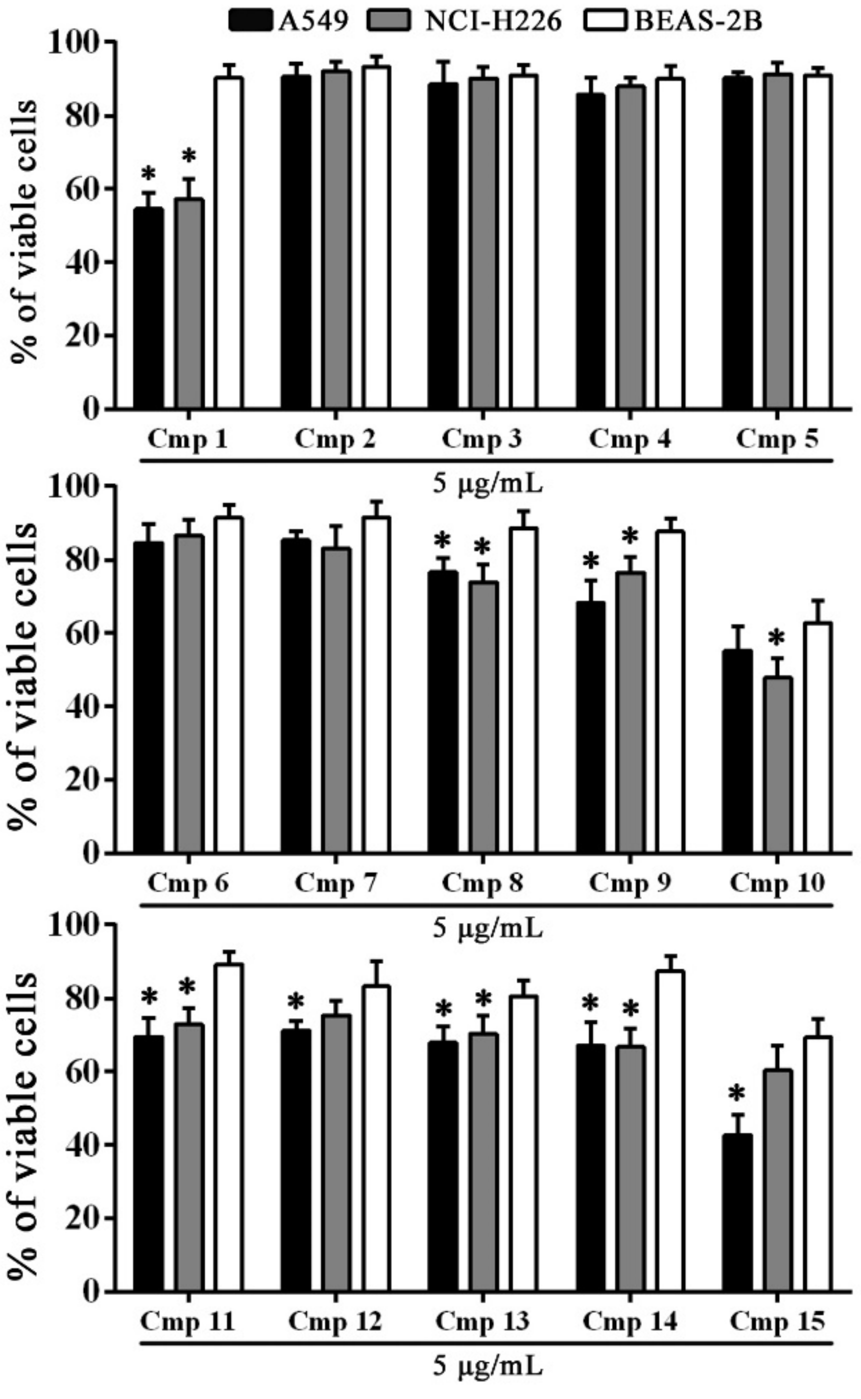

Supplementary Figure 3. Inhibitory effect of SF isolation on NSCLC cell proliferation. Cell viability was determined using trypan-blue exclusion assay. A549, NCI-H226, and BEAS-2B cells were treated with SF isolation (5 μg/mL) for 24 h. The data are shown as the means ± SD of three independent experiments. *$p < 0.01$ compared to BEAS-2B group.

References

1. Liu, C.; Liu, H.; Wen, Y.; Huang, H.; Hao, J.; Lv, Y.; Qin, R.; Yang, X. Aspernolide a inhibits the proliferation of human laryngeal carcinoma cells through the mitochondrial apoptotic and STAT3 signaling pathways. *Molecules* **2019,** *24*, 1074. doi:10.3390/molecules24061074
